# Tsr Chemoreceptor Interacts With IL-8 Provoking *E. coli* Transmigration Across Human Lung Epithelial Cells

**DOI:** 10.1038/srep31087

**Published:** 2016-08-10

**Authors:** Bing Han, Manshu Li, Yonghao Xu, Diana Islam, Julie Khang, Lorenzo Del Sorbo, Warren Lee, Katalin Szaszi, Nanshan Zhong, Arthur S. Slutsky, Yimin Li, Haibo Zhang

**Affiliations:** 1 The State Key Laboratory of Respiratory Disease, Guangzhou Institute of Respiratory Disease, and the First Affiliated Hospital of Guangzhou Medical University, Guangzhou, China; 2 Keenan Research Centre for Biomedical Science of St. Michael’s Hospital, Toronto, Canada; 3 Interdepartmental Division of Critical Care Medicine, University of Toronto, Toronto, Ontario, Canada; 4 Department of Anesthesia, University of Toronto, Toronto, Ontario, Canada; 5 Department of Physiology, University of Toronto, Toronto, Ontario, Canada

## Abstract

Bacterial colonization of epithelial surfaces and subsequent transmigration across the mucosal barrier are essential for the development of infection. We hypothesized that the methyl-accepting proteins (MCPs), known as chemoreceptors expressed on *Escherichia coli (E. coli)* bacterial surface, play an important role in mediating bacterial transmigration. We demonstrated a direct interaction between human interleukin-8 (IL-8) and Tsr receptor, a major MCP chemoreceptor. Stimulation of human lung epithelial cell monolayer with IL-8 resulted in increased *E. coli* adhesion and transmigration of the native strain (RP437) and a strain expressing only Tsr (UU2373), as compared to a strain (UU2599) with Tsr truncation. The augmented *E. coli* adhesion and migration was associated with a higher expression of carcinoembryonic antigen-related cell adhesion molecule 6 and production of inflammatory cytokines/chemokines, and a lower expression of the tight junction protein claudin-1 and the plasma membrane protein caveolin-1 in lung epithelial cells. An increased *E. coli* colonization and pulmonary cytokine production induced by the RP437 and UU2373 strains was attenuated in mice challenged with the UU2599 strain. Our results suggest a critical role of the *E. coli* Tsr chemoreceptor in mediating bacterial colonization and transmigration across human lung epithelium during development of pulmonary infections.

Pulmonary infection is frequently associated with translocation of pathogenic microorganisms across lung epithelium into the circulation and thus gain access to other organ systems^1^. Pulmonary infection is the most common cause of the acute respiratory distress syndrome (ARDS), that is associated with increased production and release of inflammatory cytokines/chemokines, such as tumor necrosis factor-α (TNF-α), interleukin-6 (IL-6), and IL-8^2^. The excessive production of cytokines/chemokines is correlated with the severity and mortality in critically ill patients^3^. Increasing evidence from experimental and clinical studies suggest that overwhelming production of cytokines/chemokines may facilitate bacterial infection. For example, *Escherichia coli (E. coli*) growth was enhanced by exposure to IL-2, granulocyte-macrophage colony-stimulating factor (GM-CSF)^4^ and IL-1^5^, and TNF-α^6^ *in vitro* and *in vivo*, respectively. Exposure to IL-1β also increased proliferation of *Staphylococcus aureus* and *Acinetobacter spp*., while *Pseudomonas aeruginosa* growth was enhanced in the presence of IL-6^7^. These findings indicate that the interaction between bacteria and cytokines^8^ may explain the high frequency of nosocomial infections in patients with an exaggerated release of cytokines^9^.

IL-8 is a major human CXCL chemokine that has been shown to be elevated locally and systemically during the inflammatory response and particularly in lung infections^10^,^11^. The present study will address whether IL-8 plays a role in bacterial colonization and transmigration.

Lung infections caused by pathogens are a complex attack system, capable of interrupting the mechanical barrier functions including mucus clearance and the innate immune system. The gram-negative bacteria *E. coli* is one of the most common pathogens found in patients with respiratory infections^12^,^13^ and sepsis^14^,^15^. It has been reported that approximately 14% of community and hospital acquired pneumonia, especially ventilator-associated pneumonia was due to *E. coli*^16^,^17^. Infection with *E. coli* is initiated by bacterial colonization and adherence to the epithelial surface and subsequent transmigration across the epithelial–endothelial barrier. Previous studies have shown that TNF-α and interferon-γ (IFN-γ) can induce *E. coli* translocation across a monolayer of intestinal epithelial cells^18^,^19^, suggesting that inflammatory cytokines may be potential chemoattractants for bacterial translocation.

The chemotactic behaviors of microorganisms are mediated by a group of methyl-accepting chemotaxis proteins (MCPs) known as chemoreceptors^20^. The MCPs are transmembrane receptors that contain different ligand-binding domains^21^. In responding to different chemotactic ligands the chemoreceptors send signals to their cytoplasmic kinase control domains, in turn driving the flagella and directing the chemotactic mobility^22^. The MCP family, including five phenotype chemoreceptors (Tsr, Tar, Tap, Trg, and Aer) expressed on *E. coli* has been most extensively characterized^20^. Among them, Tsr and Tar are the most abundantly expressed chemoreceptors making approximately 10-fold more copies than the lower abundant Tap and Trg^23^. Genetic analysis has demonstrated that Tsr and Tar are uniformly conserved in motile *E. coli* strains, while the clade of extra-intestinal pathogenic *E. coli* strains underwent ancestral loss of Trg and Tap chemoreceptors^24^. Furthermore, several uropathogenic *E. coli* strains have been found to lack Trg and Tap receptors^25^.

We thus focused the present study on an investigation testing the hypothesis that Tsr has a place in mediating *E. coli* chemotaxis in response to stimulation with IL-8 in human lung epithelial cells.

## Results

### Tsr chemoreceptor plays a critical role in the IL-8-induced *E. coli* adhesion and internalization in human lung epithelial cells

**Figure 1:**
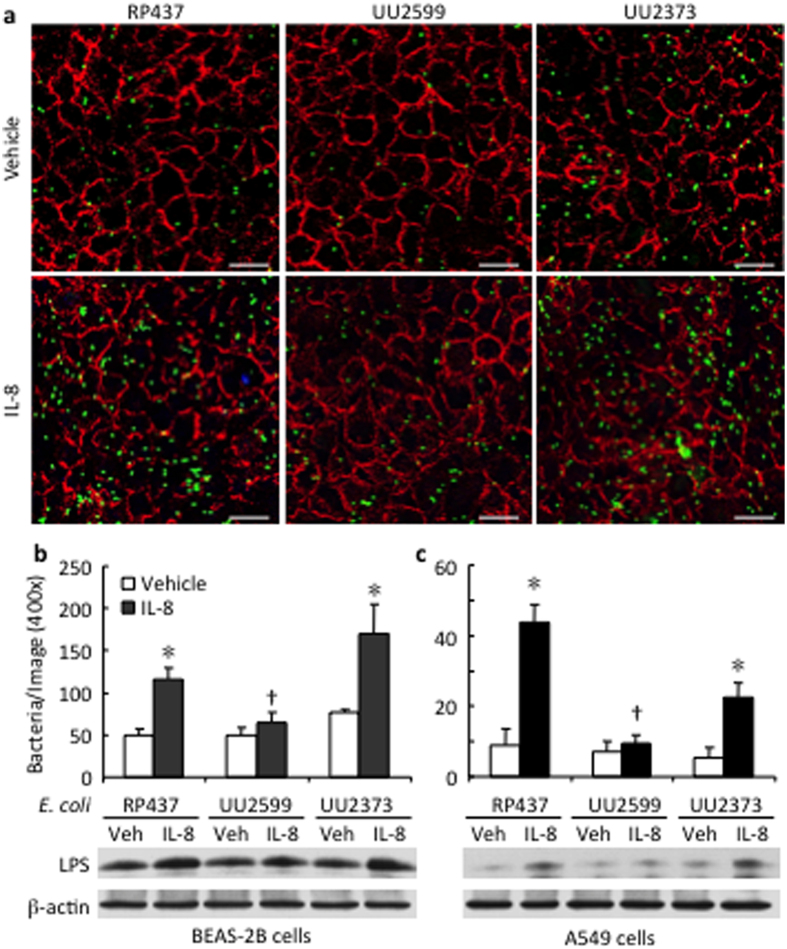
Tsr chemoreceptor mediates *E. coli* adherence to human lung epithelial cells. BEAS-2B and A549 cells were cultured on glass cover slips until fully confluent. Indicated strains of *E. coli* were added to the monolayer of the epithelial cells at same multiplicity of infection (MOI = 5) and co-cultured in absence or presence of recombinant human IL-8 (rhIL-8, 1 μg/ml). After 6 h incubation, the wells were thoroughly washed to remove the free bacteria. The BEAS-2B cells and the bacteria adhered onto or internalized into the cells were illustrated by immunofluorescence staining of E-cadherin in red and *E. coli* LPS in green (Scale bar 10 μm). (**a**) The numbers of bacteria adhered on BEAS-2B cells (**b**) and on A549 cells (**c**) were counted from 6 fields (400 x) in each condition, and are shown as mean ± SE. The adherence and internalization of *E. coli* was further confirmed by detection of LPS in the cell lysates using Western Blot. RP437: the native strain of *E. coli*; UU2599: Tsr knockout strain; UU2373: Tsr expression only strain. *p < 0.05 vs. Vehicle for the same strain, respectively; ^†^p < 0.05 vs. RP437 and UU2373 in the rhIL-8 treated-conditions, respectively.

When the *E. coli* strains were incubated with the human lung epithelial BEAS-2B cell monolayer for 6 h, the amount of bacteria (green) adhered and internalized in the epithelial cells was similar across the three strains of *E. coli* (Fig. 1a,b). However, when the *E. coli* strains were stimulated with the recombinant human IL-8 (rhIL-8), the numbers of *E. coli* adhered and internalized were higher in the native strain RP437 (116 ± 13 bacteria/field) and the strain UU2373 expressing only Tsr (170 ± 35 bacteria/field) than in the strain UU2599 lacking the Tsr gene (65 ± 12 bacteria/field, both p < 0.05) (Fig. 1a,b). Similar results were observed when the *E. coli* strains were added to human lung epithelial A549 cell monolayer (44 ± 5 bacteria/field in RP437, 23 ± 4 in UU2373, and 10 ± 2 in UU2599 (both p < 0.05, Fig. 1c). An enhanced detection of LPS was observed after stimulation with IL-8 in the cell lysates of the lung epithelial cells in co-culture with the RP437 and UU2373 strains as compared to that with the UU2599 strain (Fig. 1b,c).

### Tsr was required for the IL-8 and carcinoembryonic antigen-related cell adhesion molecule 6 (CEACAM6)-mediated *E. coli* adhesion on human lung epithelial cells

**Figure 2:**
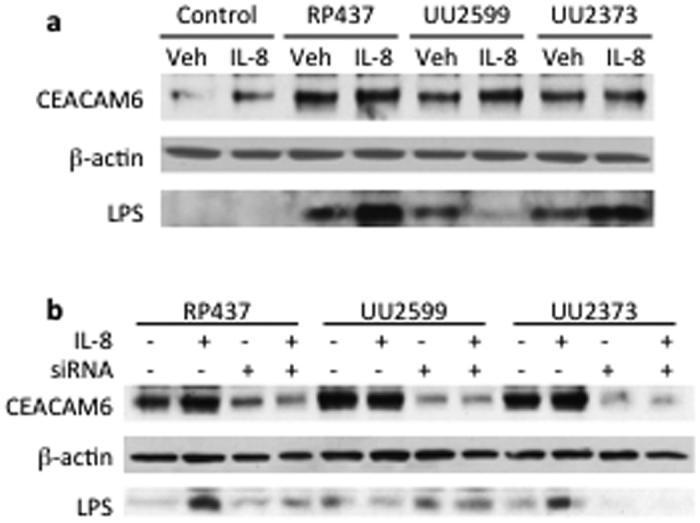
Representative illustrations of the expression and knock down of CEACAM6 in relation to bacterial adhesion in response to stimulation with IL-8 and the *E. coli* strains in human lung epithelial cells. BEAS-2B cells were treated with rhIL-8, different strains of *E. coli*, or their combination. (**a**) CEACAM6 expression and the *E. coli* LPS were determined in the cell lysates. (**b**) Effects of CEACAM knock down using siRNA on expression of CEACAM6 and LPS detection.

To examine the mechanisms by which the interaction between Tsr and IL-8 increased bacterial adhesion to human lung epithelial cells, we detected the changes of expression of CEACAM6, a receptor responsible for adherent-invasive *E. coli* in intestinal mucosa colonization^26^, after stimulation with IL-8, the three strains of *E. coli*, or their combination in BEAS-2B cells (Fig. 2a). Stimulation of the lung epithelial cells with IL-8 alone or each of the three strains of *E. coli* led to an upregulation of CEACAM6 protein level (Fig. 2a). The combination of IL-8 with either RP437 or UU2373 but not UU2599 resulted in further increased expression of CEACAM6 associated with higher bacterial adhesion and internalization as assessed by the increased LPS detection in the whole cell lysates (Fig. 2a). Knocking down the *CEACAM6* gene using specific siRNA abolished the LPS levels (Fig. 2b).

### Tsr is essential in mediating the IL-8 induced *E. coli* transmigration across human lung epithelial cells

**Figure 3:**
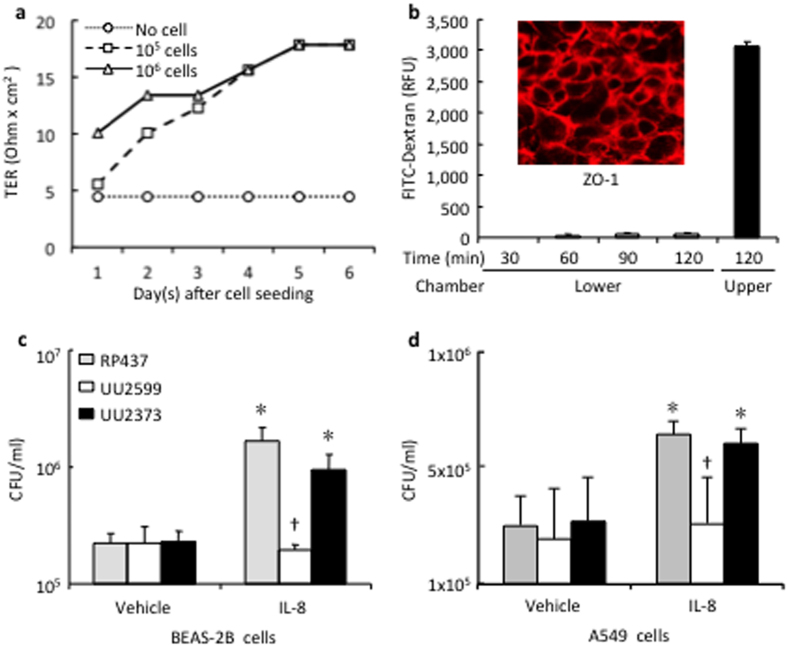
Tsr receptor mediates *E. coli* transmigration across lung epithelial cells in response to IL-8 stimulation. (**a**) BEAS-2B cells were seeded on Transwell inserts and cultured for 6 days. The monolayer formation was monitored daily by measuring transepithelial resistance (TER). (**b**) The permeability was tested by measuring fluorescence intensity in the lower chamber after loading FITC-conjugated Dextran into the upper chamber. The cell-cell tight-junction was confirmed by immunofluorescence staining (insert). (**c**,**d**) Different strains of *E. coli* were added into the upper chamber and rhIL-8 (1 μg/ml) or vehicle control was administered in the lower chamber. Bacteria transmigrated across BEAS-2B and A549 cells into the lower chamber were counted 6 h later. *p < 0.05 vs. vehicle control of the same strain, respectively (n = 3); ^†^p < 0.05 vs. RP437 and UU2373 in the IL-8 treated-conditions, respectively (n = 3).

To determine the role of the Tsr chemoreceptor on the *E. coli* bacterial chemotactic translocation across lung epithelial cell monolayer, BEAS-2B cells were cultured on a transwell system. The formation of cell monolayer and cell-cell junction was reached 5 days later at a TER plateau of 18 ohms × cm^2^ (Fig. 3a). The maturation of cell monolayer was further confirmed by the lack of leakage of FITC-Dextran (Fig. 3b). ZO-1 immunofluorescence staining showed clear cell-cell junction lines of epithelial monolayers (Fig. 3b, insert).

Using the Transwell system, administration of IL-8 in the lower chamber led to significant transmigration across the epithelial monolayer of the native strain of RP437 from 2.2 ± 0.5 in the control to 16.5 ± 5.1 × 10^5^ CFU/ml in the IL-8-treated group (p < 0.05). The chemotactic effect of IL-8 was largely attributed to the Tsr chemoreceptor since stimulation with the strain UU2373 expressing only Tsr had similar effects on *E. coli* transmigration as that stimulated with RP437 strain (Fig. 3c). On the contrary, the IL-8 mediated *E. coli* transmigration was absent in the Tsr knockout strain of UU2599 (1.9 ± 0.3 × 10^5^ CFU/ml) (Fig. 3c). Similar results were noticed when A549 cells used for the transmigration study (Fig. 3d).

### Tsr plays critical role in *E. coli* colonization in a mouse model of pneumonia

**Figure 4:**
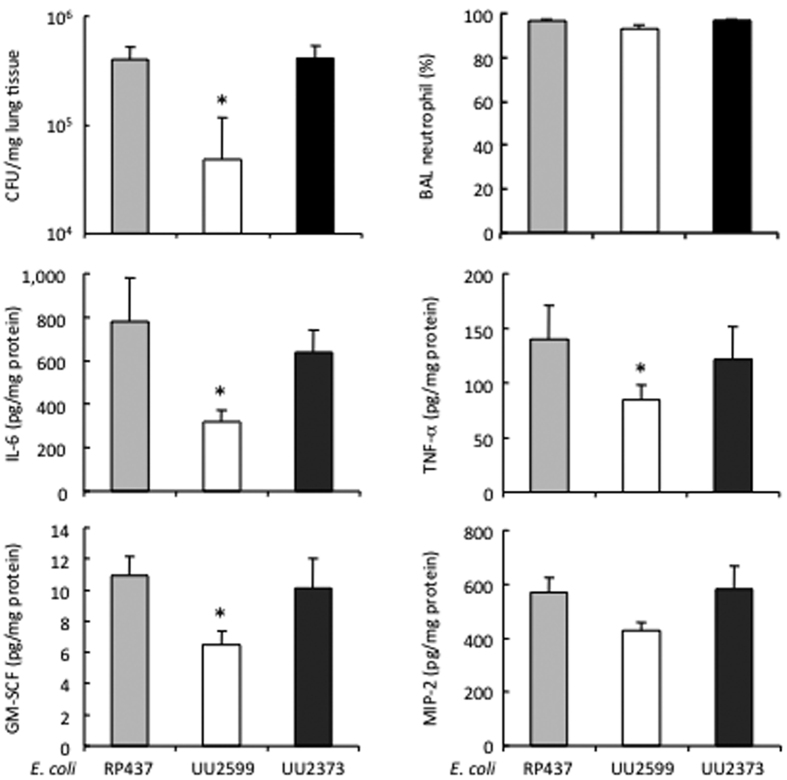
Tsr receptor mediates *E. coli* colonization and cytokine response in a mouse model of pneumonia. Mouse pulmonary infection was induced by intranasal instillation of three *E. coli* strains and observed for 24 h. The bacterial CFU counting was performed in the right lung homogenates. The neutrophil was counted in the BAL. The concentrations of IL-6, GM-CSF, TNF-α and MIP-2 were measured in lung tissue. *p < 0.05 vs. RP437 and UU2373, respectively (n = 7 to 9 mice/group).

To examine whether the *E. coli* Tsr is crucial for bacterial colonization in the lung, mice were given intranasal instillation of the three strains of *E. coli* and were observed for 24 h. A higher CFU count was noted in the mice that received the RP437 (3.9 ± 0.7 × 10^5^ CFU/mg lung tissue) and the UU2373 (4.1 ± 0.7 × 10^5^ CFU/mg lung tissue) strains than that challenged with the UU2599 strain (0.5 ± 0.3 × 10^5^ CFU/mg lung tissue, both p < 0.05), despite the fact that all the mice showed a similar concentration of MIP-2 in lung tissue (Fig. 4). The augmented *E. coli* colonization seen in the mice challenged with the RP437 and UU2373 strains was associated with a higher level of IL-6, TNF-α and GM-CSF in the lung, as compared to those given the UU2599 strain (Fig. 4).

### IL-8 induced bacterial transmigration via Tsr signal involved both paracytosis and transcytosis

**Figure 5:**
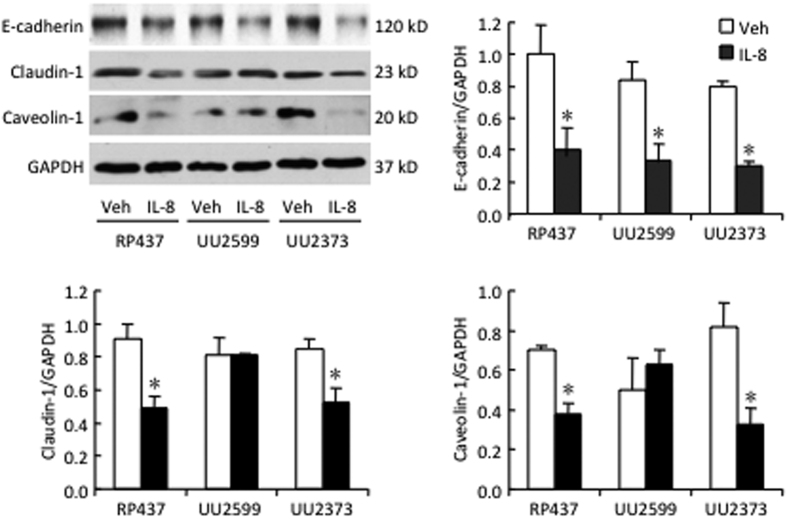
*E. coli* Tsr receptor modulates the tight junction molecule claudin-1 and plasma membrane protein caveolin-1 in human lung epithelial cells. BEAS-2B cells cultured in Transwell were inoculated with different strains of *E. coli* in upper chamber. Vehicle control or rhIL-8 (1 μg/ml) was added into the lower chamber. After 6 h incubation, expression of E-cadherin, claudin-1 and caveolin-1 in the BEAS-2B cells were determined by Western blot. Blots shown are representative from three experiments. *p < 0.05 and comparing to vehicle control (Veh) of same strain, respectively.

The stimulation with IL-8 resulted in decreased expression of the human lung epithelial cell-cell junction protein E-cadherin, responsible for paracytosis in all *E. coli* strains tested (Fig. 5). The expression of claudin-1, another protein responsible for paracytosis and caveolin-1, responsible for transcytosis decreased in the RP437 and the UU2373 strains but not in the UU2599 strain lacking Tsr gene (Fig. 5).

### Tsr mediated the IL-8-induced cytokine/chemokine responses in human lung epithelial cells by interaction with the *E. coli* bacteria

**Figure 6:**
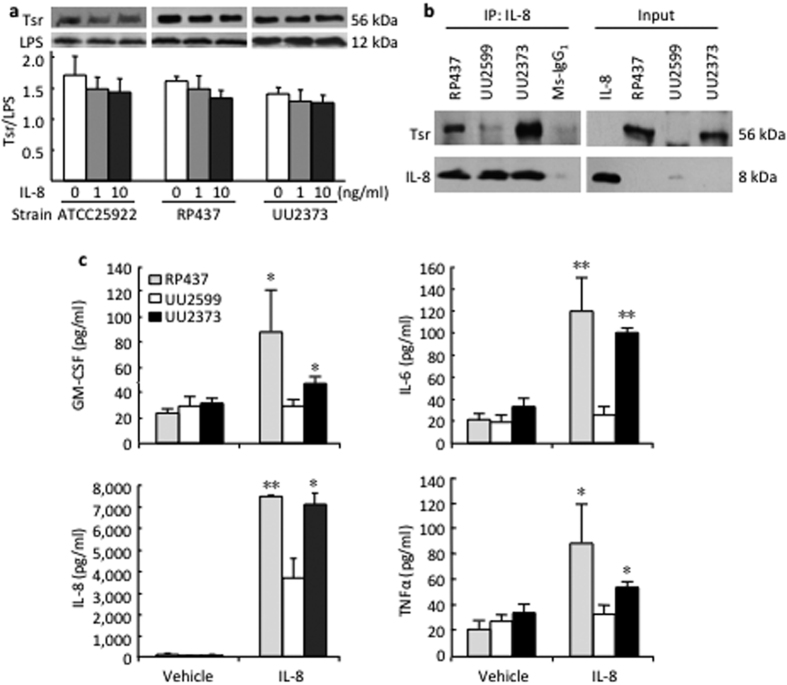
*E. coli* Tsr receptor mediates cytokine production in response to IL-8 stimulation by human lung epithelial cells. (**a**) Indicated strains of *E. coli* were cultured in absence or presence of rhIL-8 at indicated concentrations for 8 h. Tsr chemoreceptor expression was slightly decreased as detected by Western blot and quantified against LPS expression by densitometry. (**b**) Same amount (250 μg) of protein isolated from different strains of *E. coli* was incubated with IL-8. The binding complex of Tsr and IL-8 was precipitated with a monoclonal antibody against IL-8 and detected with antibodies raised against Tsr and IL-8, respectively. Recombinant human IL-8 and the *E. coli* proteins extracted from different strains were subjected to direct Western blot as input control (right panel). (**c**) Monolayer of BEAS-2B cells cultured in Transwell was inoculated with different strains of *E. coli* in the upper chamber. Vehicle control or rhIL-8 was added into the lower chamber. After 6 h incubation, multiple cytokines/chemokines in the supernatants of culture medium from the upper chambers were simultaneously measured. *p < 0.05 vs. rhIL-8 treated UU2599 (n = 3).

To examine whether there is a direct interaction between IL-8 and Tsr, two strains of native and the UU2373 *E. coli* bacteria were stimulated with rhIL-8 for 8 h, resulting in no significant change of Tsr expression (Fig. 6a). We further demonstrated direct binding between the two molecules as determined by the immunoprecipation assay (Fig. 6b).

Since inflammatory cytokines/chemokines can also contribute to increased lung epithelial permeability, we measured 10 cytokines/chemokines and observed higher levels of granulocyte-macrophage colony-stimulating factor (GM-CSF), IL-6, IL-8, and TNF-α among them assayed, in response to stimulation with IL-8 when the epithelial cells were co-cultured with RP437 strain and the UU2373 strain, as compared to the UU2599 strain lacking Tsr gene (Fig. 6c).

## Discussion

We demonstrate that the *E. coli* Tsr chemoreceptor can directly interact with human chemokine IL-8 to facilitate *E. coli* adherence leading to the bacteria transmigration across human lung epithelial cells. An enhanced inflammatory response was also observed as a result of the interaction between *E. coli* Tsr chemoreceptor and the human lung epithelial cells. Thus our study suggests that the *E. coli* Tsr chemoreceptor may serve as a therapeutic target to reduce the bacterial colonization and invasion, and the subsequent inflammatory responses in lung infection.

*E. coli* is one of the most frequent causes of many common bacterial infections documented not only in the gut, the urinary tract but also in the lung. A recent Canadian study describing the epidemiology of ICU-acquired Gram-negative bacteremia in 2004–2012 reported that the most common source of bacteremia was pneumonia (33%). Of 83 Gram-negative isolates, *E. coli* (20%) and *Pseudomonas aeruginosa* (18%) were most common^27^. Another recent French retrospective 5-year trend analysis of microbial aetiology of ICU patients with ventilator-associated pneumonia (VAP) reported that Enterobacteriaceae have overtaken *P. aeruginosa* as the leading pathogens responsible for VAP^28^. In fact, the incidence of VAP episodes due to *E. coli* increased by 76.9% in 252 episodes of VAP in 184 patients identified between 2007 and 2011^28^. We therefore focused our study on the mechanisms by which *E. coli* interacts with lung epithelial cells to seek novel therapeutic targets in lung infection.

Respiratory tract colonization is an essential step for bacterial acquisition and attachment to the epithelial lining leading to invasive lung disease. Stable colonization of bacteria requires adherence to the mucosal surface^29^. Pili have been recognized as important adhesive factors for adhesion and colonization for pneumococci^30^, *H. influenzae*^31^, and *Moraxella catarrhalis*^32^. The food-borne enterohemorrhagic *E. coli* O157:H7 produces adhesive type IV pili that are likely to contribute to intestinal colonization^33^. Since patients with VAP had infections of the *E. coli* K12 strain^34^, we examined the mechanisms on how the *E. coli* K12 interacts with human lung epithelial cells.

*E. coli* K12 has five transmembrane MCP chemoreceptor family members including Tsr, Tar, Trg, Tap and Aer^35^. These chemoreceptors are responsible for mediating chemotactic mobility of bacteria. Among them, Tsr and Tar are the most conserved and abundant chemoreceptors. Using the native RP437 strain and the UU2373 strain expressing only Tsr, as compared to the UU2599 strain lacking the Tsr gene, our results demonstrate that Tsr is the key adhesive factor for the *E. coli*-human lung epithelial cell interaction; this interaction was significantly enhanced in the presence of rhIL-8.

Colonizing bacteria pass through the mucous layer, in part facilitated by their capsule, and reach the epithelial surface and bind loosely to carbohydrates, and tightly to proteins on host surfaces. The ability of bacterial transmigration through mucous barriers is a crucial process for the development of local and systemic infection. Intestinal bacterial translocation is a recognized cause of surgical sepsis^1^,^25^. It has been shown that the pro-inflammatory cytokines interferon-γ^18^ and TNF-α^19^ can induce *E. coli* bacterial translocation in the gut. However, the mechanisms by which *E. coli* interacts with cytokines and tissues remain to be elucidated. It has been recently reported that the deletion of both Tsr and Tar in *E. coli* abolish bacterial chemotaxis to human urine, but no single deletion of either Tsr or Tar was tested in the previous study. Our data shows that the single deletion of Tsr resulted in significant attenuation of the IL-8 induced bacterial adherence and transmigration in human lung epithelial cells, and decreased *E. coli* colonization and cytokine responses in a mouse model of pneumonia.

There are two major mechanisms, including paracytosis (i.e., claudin-1 and E-cadherin-facilitated cell-cell junction integrity)^36^,^37^ and transcytosis (i.e, caveolae-mediated internalization)^38^ that are responsible for microorganism transmigration or invasion of mucous barrier. Our results demonstrated that that IL-8 and bacteria interaction regulates the expression of cell-cell junction proteins (E-cadherin and cludin-1) as well as caveolae protein (caveolin-1) in the lung epithelial cells. Therefore, both paracytosis and transcytosis are involved in the Tsr-mediated *E. coli* transmigration by increasing the permeability in human lung epithelial cells. In addition, there is increasing evidence indicating that cytokines and other cellular factors can mediate a loss of cellular barrier function^39^. It has been shown that lung epithelial cells are not only the target of inflammatory insults, but also the active contributors to the inflammatory responses^40^. We observed that Tsr plays an important role in mediating the *E. coli*-induced cytokine and chemokine responses in human lung epithelial cells. This observation may explain how *E. coli* bacterial invasion causes inflammatory responses during lung infection. Enhanced production of inflammatory cytokines and chemokines is a fundamental component of the host defense system in innate immunity to help (directly and indirectly) eradicate bacteria at the site of infection. However, when excessive inflammatory responses take place, this primary defense process may instead facilitate bacterial infection, which in turn prolongs the inflammatory responses leading to organ failure. In addition, an overwhelming cytokine/chemokine response has been suggested to contribute to damage pulmonary blood gas barrier and thus may be a potential therapeutic target for lung injury^41^.

It has been reported that adhesion of certain *E. coli* depends on expression of CEACAM6 in small intestinal cells^26^,^42^. We observed that stimulation with either IL-8 alone or the *E. coli* strain each used was able to increase the expression of CEACAM6 and bacterial adhesion and internalization independent of Tsr signal in lung epithelial cells. However, the synergic effects of IL-8 and *E. coli* on bacterial adhesion seen in RP437 and UU2373 strains was lost in the UU2599 strain, suggesting that Tsr is required for the CEACAM6-mdeiated *E. coli* adhesion.

In summary, our study suggests a critical role of the *E. coli* Tsr chemoreceptor in mediating bacterial colonization and transmigration in human lung mucosal barrier. *E. coli* Tsr may serve as a novel therapeutic target to detain the development of pulmonary infection.

## Methods

### Bacterial strains

All bacterial strains used in this study were *E. coli* K-12 strain RP437 and its derivatives provided by Dr. Parkinson of University of Utah^43^. RP437 is a standard native *E. coli* strain; it has all of the chemoreceptors. The other strains used are RP437 derivatives: UU2599 has all chemoreceptors except for Tsr [(*tsr*)∆5547], and UU2373 has only Tsr with other chemoreceptors deleted [(*tar-tap*)∆5201 (*aer*)∆1 ygjG::Gm (*trg*)∆4543]^43^.

All the *E. coli* strains were streaked on Columbia blood agar plates with 5% sheep blood and incubated at 37 °C overnight to revive. Colonies from the three strains were picked and grown in Tryptic Soy Broth (TSB, 5 ml per colony) at 37 °C with 170 rpm shaking (MaxQ^TM^ 4500 orbital shaker, Thermo Scientific, Midland, ON, Canada) overnight.

### Tsr expression and its interaction with IL-8

To examine the potential effect of IL-8 on Tsr expression, wild type *E. coli* strains (ATCC25922 and RP437) and the strain expression only Tsr (UU2373) at 10^5^ CFU/ml was cultured with or without recombinant human IL-8 (rhIL-8, 1 and 10 ng/ml, Invitrogen, Burlington, ON, Canada) at 37 °C for 8 h in a shaking incubator. The protein expression of Tsr from bacterial culture in the presence or absence of IL-8 was detected with Western blot using an anti-Tsr antiserum (gift from Dr. Parkinson, University of Utah).

The direct interaction between Tsr and IL-8 was detected by co-immunoprecipitation. Briefly, the bacteria were collected (1 ml/strain) from the bacterial suspensions (0.5 at OD_600nm_) by centrifugation at 4 °C, 10,000 g for 5 min (Microfuge 22R, Beckman Coulter, Mississauga, ON, Canada). The bacterial pellets were washed with PBS and lysed in a cell lysis buffer containing 1% Triton X-100 and protease inhibitors. The same amount of protein (250 μg) from each strain was incubated with rhIL-8 (0.5 μg) and mouse monoclonal anti-human IL-8 antibody (1 μg, R&D System, Minneapolis, MN) at 4 °C overnight while being rotated (Fisher Scientific Labquake 415110Q, Ottawa, ON). The immune-complexes were precipitated by incubation with Protein A/G UltraLink® Resin (Pierce Biotechnology, Rockford, lL) and then subjected to Western blotting with the antibodies against Tsr and IL-8.

### Human lung epithelial cell culture

Human bronchial epithelial cells BEAS-2B (ATCC CRL-9609; Manassas, VA) and human alveolar type II-like epithelial A549 cells (ATCC CCL-185) were cultured in Dulbecco’s modified Eagle’s medium (DMEM, Sigma, St. Louis, MA) supplemented with 10% fetal bovine serum (FBS, Sigma), 100 U/ml penicillin and 100 μg/ml streptomycin.

### Bacterial adhesion and internalization to human lung epithelial cells

To determine the adherent ability of different strains of *E. coli* to BEAS-2B and A549 cells, the cells were cultured to confluence on glass cover slips in 24-well plates. The culture medium was removed and the cells were rinsed with PBS before bacterial infection. Indicated strains of the *E. coli* (2 × 10^6^ CFU/ml) were prepared in DMEM without FBS and antibiotics, and added onto the BEAS-2B or A549 cell monolayer at a multiplicity of infection (MOI) of 5–10, and then incubated at 37 °C in the absence or presence of rhIL-8 (1 μg/ml) for 2–8 h. The culture medium was removed and the culture plates were thoroughly washed 3 times with PBS to remove unbound bacteria. The attached cells were fixed with 4% paraformaldehyde for 10 min followed by 3 washes with PBS, and then permeabilized with 0.025% Triton X-100 for 3 min at room temperature for immunofluorescence staining. Non-specific binding was blocked with 5% bovine serum albumin (BSA, Sigma) dissolved in PBS containing 0.1% Tween-20 (PBST). The bacteria adhered/internalized to the cells were stained with a goat polyclonal antibody against *E. coli* specific LPS (AbD Serotec, Raleigh, NC) and illustrated in green with Alexa488-conjugated bovine anti-goat IgG (Jackson ImmunoResearch Laboratory, West Grove, PA). The epithelial cell monolayer was shown by staining with a mouse monoclonal antibody against E-cadherin (Santa Cruz Biotechnology, Santa Cruz, CA) and illustrated in red using Cy3-conjugated donkey anti-mouse IgG (Jackson ImmunoResearch). The immunofluorescence images were taken and analyzed under a Zeiss LSM-700 confocal microscope (Zeiss, Toronto, ON, Canada). Since we observed that the adherence peaked at 6 h after bacterial infection in co-culture with the lung epithelial cells, the subsequent studies focused on 6-h timing and MOI of 5 unless otherwise indicated.

In a set of parallel experiments, whole cell lysates were collected from BEAS-2B cells receiving the same *E. coIi* and IL-8 treatment for determining LPS with Western blotting.

### Protein expression and gene knock down of CEACAM6

CEACAM6 expression was detected in BEAS-2B cells after treatment with IL-8, different strains of *E. coli*, or the combination of IL-8 and the strains respectively for 6 h. The *CEACAM6* gene was knocked down using a siRNA against *CEACAM6* (ON-TARGETPlus human CEACAM6 siRNA, GE Dharmacon Health Care Inc., Burlington, NC) for 48 h, and then treated with *E. coli* and IL-8 as described above. The whole cell lysates were subjected to Western blot and probed with specific antibodies against LPS (AbD Serotec) and CEACAM6 (Abcam, Toronto, Canada).

### Bacterial translocation across human lung epithelial cells

To assess bacterial translocation across lung epithelial cell monolayer, a transwell system (Transwell culture inserts with 1.12 cm^2^ growth area and 3 μm pore size, Costar, Corning, NY) was used. Different numbers (10^5^ and 10^6^ cells/well) of BEAS-2B cells were seeded on the filter membrane of the upper chamber insert and were cultured for 6 days to allow polarization and formation of cell-cell junctions. The transepithelial resistance (TER) was measured with an Epithelial Voltohmmeter (EndOhm, World Precision Instrument, Sarasota, FL) to determine the maturity of cell monolayer and cell-cell junction. FITC-conjugated Dextran (Molecular probe, Eugene, OR) was added into the upper chambers of the Transwell and any potential leakage of FITC-Dextran was monitored for 2 h by measuring fluorescence in medium from the lower chamber. Finally the filter with cells was removed from the insert holder for immunofluorescence staining of ZO-1 as described above to demonstrate the integrity of tight junction of the cell monolayer.

All subsequent bacterial translocation experiments were done on the BEAS-2B and A549 cell monolayer cultured for 6 days on the permeable filter with a TER stabilized at 18 ohm × cm^2^. Different strains of *E. coli* were prepared in DMEM without FBS and antibiotics, and added onto the apical side of the cell monolayer in the upper chamber of the Transwell (MOI = 10); and rhIL-8 was administered into the basal medium (FBS- and antibiotics-free) in the lower chamber of the Transwell and co-cultured for 6 h. The medium from both chambers of the Transwells, as well as whole cell lysate were collected for further analysis by Western blots and ELISAs. The bacterial translocation across the lung epithelial cells into the medium collected from the lower chamber was determined by CFU counting as previously described^6^.

### Mouse model of pulmonary *E. coli* infection

All experimental protocols were approved by the Animal Care Committee of the St. Michael’s Hospital, and were performed in accordance with the Canadian Council on Animal Care guidelines and regulations. Briefly, male C57BL/6 mice (12–14 weeks, and body weight of 28–32 g, Jackson Laboratories, Bar Harbor, ME) were anesthetized with ketamine hydrochloride (Wyeth-Ayerst, Mississauga, ON, Canada) and xylazine (Bayer, Mississauga, ON, Canada) as we have previously reported^40^. The mice were then randomized to receive intranasal instillation of 50 μl of 2 × 10^7^ CFU of the three strains of *E. coli,* respectively. The PBS solution was used to serve as a vehicle control group. The animals were then observed for 24 h, and bronchoalveolar lavage (BAL) was collected under anesthesia by bolus injection of 0.8 ml PBS into the lung 3 times. The BAL fluid was collected and cytospins were performed for neutrophil counting. The right and left lungs were dissected and homogenized separately. The right lung tissue was for bacterial CFU counting and the left lung tissue was for measurement of macrophage inflammatory protein (MIP)-2, the murine IL-8 homologue.

### Cell tight junction protein assays

The BEAS-2B cells on the filter of the Transwell inserts were washed twice with ice cold PBS, and lysed by incubating with PBS containing 1% Triton X-100 and protease inhibitors. The whole cell lysates were ran on 10% SDS-PAGE and transferred on nitrocellulose membranes for Western blotting with a rabbit polyclonal antibody against human claudin-1 (Invitrogen, Camarillo, CA) and mouse monoclonal antibodies against human E-cadherin (Santa Cruz biotechnology) and human caveolin-1 (BD Science, Mississauga, ON).

### Measurement of cytokines/chemokines

The medium collected from upper chambers of the transwell and the left lung homogenates were centrifuged at 4 °C 18,000 g for 10 min. The concentrations of multiple human and mouse cytokines/chemokines in the supernatants were measured using the ProcartaPlex Human and mouse Basic Kits, respectively (e-Bioscience/Affymetrix, Santa Clara, CA).

### Statistical Analysis

Results are expressed as mean ± SEM. The significance of the differences was analyzed by an unpaired *t* test or a one-way ANOVA, using the Bonferroni or Dunn test for comparisons of two or more groups. P < 0.05 was considered to be significant.

## Additional Information

**How to cite this article**: Han, B. *et al.* Tsr Chemoreceptor Interacts With IL-8 Provoking *E. coli* Transmigration Across Human Lung Epithelial Cells. *Sci. Rep.* **6**, 31087; doi: 10.1038/srep31087 (2016).
